# A general theorem on the stability of a class of functional equations including quadratic-additive functional equations

**DOI:** 10.1186/s40064-016-1771-y

**Published:** 2016-02-24

**Authors:** Yang-Hi Lee, Soon-Mo Jung

**Affiliations:** Department of Mathematics Education, Gongju National University of Education, Gongju, 32553 Republic of Korea; Mathematics Section, College of Science and Technology, Hongik University, Sejong, 30016 Republic of Korea

**Keywords:** Generalized Hyers-Ulam stability, Functional equation, *n*-dimensional quadratic-additive type functional equation, Direct method, 39B82, 39B52

## Abstract

We prove a general stability theorem of an *n*-dimensional quadratic-additive type functional equation

$$\begin{aligned} Df(x_1, x_2, \ldots , x_n) = \sum _{i=1}^m c_i f \big ( a_{i1}x_1 + a_{i2}x_2 + \cdots + a_{in}x_n \big ) = 0 \end{aligned}$$

by applying the direct method.

## Background

Throughout this paper, let *V* and *W* be real vector spaces, let *X* and *Y* be a real normed space and a real Banach space, respectively, and let ${\mathbb {N}}_0$ denote the set of all nonnegative integers. For any mapping $f : V \rightarrow W$, let us define

$$\begin{aligned} f_o(x)&\,:=\, \frac{f(x)-f(-x)}{2}, \\ f_e(x)&\,:=\, \frac{f(x)+f(-x)}{2}, \\ Af(x,y)&\,:=\, f(x+y) - f(x) - f(y), \\ Qf(x,y)&\,:=\, f(x+y) + f(x-y) - 2f(x) - 2f(y) \end{aligned}$$

for all $x, y \in V$. A mapping $f : V \rightarrow W$ is called an additive mapping (or a quadratic mapping) if *f* satisfies the functional equation $Af(x,y) = 0$ (or $Qf(x,y) = 0$) for all $x, y \in V$. We notice that the mappings $g, h : {\mathbb {R}} \rightarrow {\mathbb {R}}$ given by $g(x) = ax$ and $h(x) = ax^2$ are solutions of $Ag(x,y) = 0$ and $Qh(x,y) = 0$, respectively.

A mapping $f : V \rightarrow W$ is called a quadratic-additive mapping if and only if *f* is represented by the sum of an additive mapping and a quadratic mapping. A functional equation is called a quadratic-additive type functional equation if and only if each of its solutions is a quadratic-additive mapping. For example, the mapping $f(x) = ax^2 + bx$ is a solution of the quadratic-additive type functional equation.

In the study of stability problems of quadratic-additive type functional equations, we follow out a routine and monotonous procedure for proving the stability of the quadratic-additive type functional equations under various conditions. We can find in the books (Cho et al. 2013; Czerwik 2002; Hyers et al. 1998; Jung 2011) a lot of references concerning the Hyers-Ulam stability of functional equations (see also Alotaibi and Mohiuddine 2012; Aoki 1950; Baker 2005; Brillouët-Belluot et al. 2012; Găvruţa 1994; Hyers 1941; Mohiuddine 2009; Mohiuddine and Şevli 2011; Mursaleen and Mohiuddine 2009; Rassias 1978; Ulam 1960).

In this paper, we prove a general stability theorem that can be easily applied to the (generalized) Hyers-Ulam stability of a large class of functional equations of the form $Df(x_1, x_2, \ldots , x_n) = 0$, which includes quadratic-additive type functional equations. In practice, given a mapping $f :V \rightarrow W$, $Df : V^n \rightarrow W$ is defined by

1 $$\begin{aligned} Df(x_1, x_2, \ldots , x_n) := \sum _{i=1}^m c_i f \big ( a_{i1}x_1 + a_{i2}x_2 + \cdots + a_{in}x_n \big ) \end{aligned}$$

for all $x_1, x_2, \ldots , x_n \in V$, where *m* is a positive integer and $c_i, a_{ij}$ are real constants. Indeed, this stability theorem can save us much trouble of proving the stability of relevant solutions repeatedly appearing in the stability problems for various functional equations including the quadratic functional equations (Jun and Lee 2001), the additive functional equations (Forti 2007; Lee and Jun 2000; Nakmahachalasint 2007a), and the quadratic-additive type functional equations (see Chang et al. 2003; Eskandani et al. 2012; Jun and Kim 2004a, b, 2005, 2006; Jung 1998; Jung and Sahoo 2002; Lee 2013; Nakmahachalasint 2007b; Najati and Moghimi 2008; Piszczek and Szczawińska 2013; Towanlong and Nakmahachalasint 2009).

It should be remarked that Bahyrycz and Olko (2015) applied the fixed point method to investigate the generalized Hyers-Ulam stability of the general linear equation

$$\begin{aligned} \sum _{i=1}^m A_i \left( \sum _{i=1}^n a_{ij} x_j \right) + A = 0. \end{aligned}$$

Moreover, there are numerous recent results concerning the Hyers-Ulam stability of some particular cases of the equation $Df(x_1, x_2, \ldots , x_n) = 0$. Some of them have been described in the survey paper (Brzdȩk and Ciepliński 2013).

## Preliminaries

We now introduce a lemma from the paper [Lee and Jung (2015), Corollary 2].

### **Lemma 1**

*Let *$k > 1$* be a real constant, let *$\phi : V \backslash \{ 0 \} \rightarrow [0,\infty )$*be a function satisfying either*

2 $$\begin{aligned} \Phi (x) := \sum _{i=0}^\infty \frac{1}{k^i} \phi (k^i x) < \infty \end{aligned}$$

*for all *$x \in V \backslash \{ 0 \}$*or*

3 $$\begin{aligned} \Phi (x) := \sum _{i=0}^\infty k^{2i} \phi \bigg ( \frac{x}{k^i} \bigg ) < \infty \end{aligned}$$

*for all *$x \in V \backslash \{ 0 \}$*, and let *$f : V \rightarrow Y$* be an arbitrarily given mapping. If there exists a mapping *$F : V \rightarrow Y$* satisfying*

4 $$\begin{aligned} \Vert f(x) - F(x) \Vert \le \Phi (x) \end{aligned}$$

*for all *$x \in V \backslash \{ 0 \}$* and*

5 $$\begin{aligned} F_e(kx) = k^2 F_e(x), \quad F_o(kx) = kF_o(x) \end{aligned}$$

*for all *$x \in V$*, then F is a unique mapping satisfying (*4*) and (*5*)*.

We introduce a lemma that is the same as [Lee and Jung (2015), Corollary 3].

### **Lemma 2**

*Let *$k > 1$* be a real number, let *$\phi , \psi : V \backslash \{ 0 \} \rightarrow [0,\infty )$* be functions satisfying each of the following conditions*

$$\begin{aligned} \begin{array}{lll} {\displaystyle \sum _{i=0}^\infty k^i \psi \bigg ( \frac{x}{k^i} \bigg ) < \infty }, \quad {\displaystyle \sum _{i=0}^\infty \frac{1}{k^{2i}} \phi (k^i x) < \infty },\\ {\displaystyle \tilde{\Phi }(x) := \sum _{i=0}^\infty k^i \phi \bigg ( \frac{x}{k^i} \bigg ) < \infty }, \quad {\displaystyle \tilde{\Psi }(x) := \sum _{i=0}^\infty \frac{1}{k^{2i}} \psi (k^i x) < \infty } \end{array} \end{aligned}$$

*for all *$x \in V \backslash \{ 0 \}$*, and let *$f : V \rightarrow Y$* be an arbitrarily given mapping. If there exists a mapping*$F : V \rightarrow Y$* satisfying the inequality*

6 $$\begin{aligned} \Vert f(x) - F(x) \Vert \le \tilde{\Phi }(x) + \tilde{\Psi }(x) \end{aligned}$$

for all $x \in V \backslash \{ 0 \}$* and the conditions in (*5*) for all*$x \in V$*, then F is a unique mapping satisfying (*5*) and (*6*)*.

## Main results

In this section, let *a* be a real constant such that $a \not \in \{ -1, 0, 1 \}$.

### **Theorem 1**

*Let n be a fixed integer greater than 1, let *$\mu , \nu : V \backslash \{ 0 \} \rightarrow [0,\infty )$* be functions satisfying the conditions*

7 $$\begin{aligned} \sum _{i=0}^\infty \frac{\mu (a^i x)}{a^{2i}} < \infty \quad { \text {and} \ }\quad \sum _{i=0}^\infty \frac{\nu (a^i x)}{|a|^i} < \infty \end{aligned}$$

*for all *$x \in V \backslash \{ 0 \}$*, and let *$\varphi : (V \backslash \{ 0 \})^n \rightarrow [0,\infty )$* be a function satisfying the conditions*

8 $$\begin{aligned} \sum _{i=0}^\infty \frac{\varphi (a^i x_1, a^i x_2, \ldots , a^i x_n)}{a^{2i}} < \infty \quad { \text {and} \ }\quad \sum _{i=0}^\infty \frac{\varphi (a^i x_1, a^i x_2, \ldots , a^i x_n)}{|a|^i} < \infty \end{aligned}$$

*for all *$x_1, x_2, \ldots , x_n \in V \backslash \{ 0 \}$*. If a mapping *$f : V \rightarrow Y$*satisfies *$f(0) = 0$,

9 $$\begin{aligned} \Vert f_e(ax) - a^2 f_e(x) \Vert \le \mu (x) \quad { \text {and} \ }\quad \Vert f_o(ax) - af_o(x) \Vert \le \nu (x) \end{aligned}$$

*for all *$x \in V \backslash \{ 0 \}$*, and*

10 $$\begin{aligned} \Vert Df(x_1, x_2, \ldots , x_n) \Vert \le \varphi (x_1, x_2, \ldots , x_n) \end{aligned}$$

*for all *$x_1, x_2, \ldots , x_n \in V \backslash \{ 0 \}$*, then there exists a unique mapping *$F : V \rightarrow Y$* such that*

11 $$\begin{aligned} DF(x_1, x_2, \ldots , x_n) = 0 \end{aligned}$$

*for all *$$x_1, x_2, \ldots , x_n \in V \backslash \{ 0 \}$$,

12 $$\begin{aligned} F_e(ax) = a^2 F_e(x) \quad { \text {and} \ }\quad F_o(ax) = aF_o(x) \end{aligned}$$

*for all *$x \in V$*, and*

13 $$\begin{aligned} \Vert f(x) - F(x) \Vert \le \sum _{i=0}^{\infty } \bigg ( \frac{\mu (a^i x)}{a^{2i+2}} + \frac{\nu (a^i x)}{|a|^{i+1}} \bigg ) \end{aligned}$$

*for all *$$x \in V \backslash \{ 0 \}$$.

### *Proof*

First, we define $A := \{ f : V \rightarrow Y \,|\, f(0) = 0 \}$ and a mapping $J_m : A \rightarrow A$ by

$$\begin{aligned} J_m f(x) = \frac{f_e(a^m x)}{a^{2m}} + \frac{f_o(a^m x)}{a^m} \end{aligned}$$

for $x \in V$ and $m \in {\mathbb {N}} \cup \{ 0 \}$. It follows from (9) that

14 $$\begin{aligned} \Vert J_mf(x) - J_{m+l}f(x) \Vert \le \sum _{i=m}^{m+l-1}&\Vert J_if(x) - J_{i+1}f(x) \Vert \nonumber \\ = \sum _{i=m}^{m+l-1}&\bigg \Vert \frac{f_e (a^i x)}{a^{2i}} + \frac{f_o (a^i x)}{a^i} - \frac{f_e (a^{i+1} x)}{a^{2i+2}} - \frac{f_o (a^{i+1} x)}{a^{i+1}} \bigg \Vert \nonumber \\ = \sum _{i=m}^{m+l-1}&\bigg \Vert -\frac{1}{a^{i+1}} \bigg ( f_o (a \cdot a^i x) - af_o (a^i x) \bigg ) \nonumber \\&\quad -\frac{1}{a^{2i+2}} \bigg ( f_e (a \cdot a^i x) - a^2 f_e (a^i x) \bigg ) \bigg \Vert \nonumber \\ \le \sum _{i=m}^{m+l-1}&\bigg ( \frac{\mu (a^i x)}{a^{2i+2}} + \frac{\nu (a^i x)}{|a|^{i+1}} \bigg ) \end{aligned}$$

for all $x \in V \backslash \{ 0 \}$. In view of (7) and (14), the sequence $\{ J_m f(x) \}$ is a Cauchy sequence for all $x \in V \backslash \{ 0 \}$. Since *Y* is complete and $f(0) = 0$, the sequence $\{ J_m f(x) \}$ converges for all $x \in V$. Hence, we can define a mapping $F : V \rightarrow Y$ by

$$\begin{aligned} F(x) := \lim _{m \rightarrow \infty } J_m f(x) = \lim _{m \rightarrow \infty } \bigg ( \frac{f_e (a^m x)}{a^{2m}} + \frac{f_o (a^m x)}{a^m} \bigg ) \end{aligned}$$

for all $x \in V$. We easily obtain from the definition of *F* and (10) that the equalities in (12) hold for all $x \in V$, and by (1) and (8), we get

$$\begin{aligned} \Vert DF&(x_1, x_2, \ldots , x_n) \Vert \\ =&\ \lim _{m \rightarrow \infty } \bigg \Vert \frac{Df_e \big ( a^m x_1, a^m x_2, \ldots , a^m x_n \big )}{a^{2m}} + \frac{Df_o \big ( a^m x_1, a^m x_2, \ldots , a^m x_n \big )}{a^m} \bigg \Vert \\ \le&\ \lim _{m \rightarrow \infty } \Bigg ( \frac{\varphi \big ( a^m x_1, a^m x_2, \ldots , a^m x_n \big ) + \varphi \big ( -a^m x_1, -a^m x_2, \ldots , -a^m x_n \big )}{2a^{2m}} \\&\quad \qquad +\frac{\varphi \big ( a^m x_1, a^m x_2, \ldots , a^m x_n \big ) + \varphi \big ( -a^m x_1, -a^m x_2, \ldots , -a^m x_n \big )}{2|a|^m} \Bigg ) \\ =&\ 0 \end{aligned}$$

for all $x_1, x_2, \ldots , x_n \in V \backslash \{ 0 \}$, *i.e.*, $DF(x_1, x_2, \ldots , x_n) = 0$ for all $x_1, x_2, \ldots , x_n \in V \backslash \{ 0 \}$. Moreover, if we put $m = 0$ and let $l \rightarrow \infty$ in (14), then we obtain the inequality (13).

Notice that the equalities

$$\begin{aligned} F_e(|a|x) \,=\, &|a|^2F_e(x),&F_e \bigg ( \frac{x}{|a|} \bigg ) = \frac{F_e(x)}{|a|^2}, \nonumber \\ F_o(|a|x) \,=\, &|a| F_o(x),&F_o \bigg ( \frac{x}{|a|} \bigg ) = \frac{F_o(x)}{|a|} \end{aligned}$$

are true in view of (12).

When $|a| > 1$, in view of Lemma 1, there exists a unique mapping $F : V \rightarrow Y$ satisfying the equalities in (12) and the inequality (13), since the inequality

$$\begin{aligned} \Vert f(x) - F(x) \Vert \le&\ \sum _{i=0}^{\infty } \bigg ( \frac{\mu (a^i x)}{a^{2i+2}} + \frac{\nu (a^i x)}{|a|^{i+1}} \bigg ) \\ =&\ \sum _{i=0}^{\infty } \bigg ( \frac{\mu (a^{2i}ax)}{a^{4i+4}} + \frac{\mu (a^{2i}x)}{a^{4i+2}} + \frac{\nu (a^{2i}ax)}{a^{2i+2}} + \frac{\nu (a^{2i}x)}{|a|^{2i+1}} \bigg ) \\ \le&\ \sum _{i=0}^{\infty } \frac{\phi (a^{2i}x)}{a^{2i}} \\ =&\ \sum _{i=0}^{\infty } \frac{\phi (k^i x)}{k^i} \end{aligned}$$

holds for all $x \in V$, where $k = a^2$ and $\phi (x) = \frac{\mu (x)}{a^2} + \frac{\mu (ax)}{a^4} + \frac{\nu (x)}{|a|} + \frac{\nu (ax)}{a^2}$.

When $|a| < 1$, in view of Lemma 1, there exists a unique mapping $F : V \rightarrow Y$ satisfying the equalities in (12) and the inequality (13), since the inequality

$$\begin{aligned} \Vert f(x) - F(x) \Vert \le&\ \sum _{i=0}^{\infty } \bigg ( \frac{\mu (a^i x)}{a^{2i+2}} + \frac{\nu (a^i x) }{|a|^{i+1}} \bigg ) \\ =&\ \sum _{i=0}^{\infty } \bigg ( \frac{\mu (a^{2i}x)}{a^{4i+2}} + \frac{\mu (a^{2i}ax)}{a^{4i+4}} + \frac{\nu (a^{2i}x)}{|a|^{2i+1}} + \frac{\nu (a^{2i}ax)}{a^{2i+2}} \bigg ) \\ \le&\ \sum _{i=0}^{\infty } \frac{\phi (a^{2i}x)}{a^{4i}} \\ =&\ \sum _{i=0}^{\infty } k^{2i} \phi \bigg ( \frac{x}{k^i} \bigg ) \end{aligned}$$

holds for all $x \in V$, where $k = \frac{1}{a^2}$ and $\phi (x) = \frac{\mu (x)}{a^2} + \frac{\mu (ax)}{a^4} + \frac{\nu (x)}{|a|} + \frac{\nu (ax)}{a^2}$. $\square$

### **Theorem 2**

*Let n be a fixed integer greater than 1, let *$\mu , \nu : V \backslash \{ 0 \} \rightarrow [0,\infty )$* be functions satisfying the conditions*

15 $$\begin{aligned} \sum _{i=0}^\infty |a|^i \nu \bigg ( \frac{x}{a^i} \bigg ) < \infty {\quad \text {and} \ }\quad \sum _{i=0}^\infty a^{2i} \mu \bigg ( \frac{x}{a^i} \bigg ) < \infty \end{aligned}$$

*for all *$x \in V \backslash \{ 0 \}$*, and let *$\varphi : (V \backslash \{ 0 \})^n \rightarrow [0,\infty )$* be a function satisfying the conditions*

16 $$\begin{aligned} \sum _{i=0}^\infty |a|^i \varphi \bigg ( \frac{x_1}{a^i}, \frac{x_2}{a^i}, \ldots , \frac{x_n}{a^i} \bigg ) < \infty {\quad \text {and} \ }\quad \sum _{i=0}^\infty a^{2i} \varphi \bigg ( \frac{x_1}{a^i}, \frac{x_2}{a^i}, \ldots , \frac{x_n}{a^i} \bigg ) < \infty \end{aligned}$$

*for all *$x_1, x_2, \ldots , x_n \in V \backslash \{ 0 \}$*. If a mapping *$f : V \rightarrow Y$*satisfies *$f(0) = 0$*, (*9*) for all *$x \in V \backslash \{ 0 \}$*, as well as (*10*) for all *$x_1, x_2, \ldots , x_n \in V \backslash \{ 0 \}$*, then there exists a unique mapping *$F : V \rightarrow Y$* satisfying (*11*) for all *$x_1, x_2, \ldots , x_n \in V \backslash \{ 0 \}$*, and (*12*) for all *$x \in V$*, and such that*

17 $$\begin{aligned} \Vert f(x) - F(x) \Vert \le \sum _{i=0}^{\infty } \bigg ( a^{2i} \mu \bigg ( \frac{x}{a^{i+1}} \bigg ) + |a|^i \nu \bigg ( \frac{x}{a^{i+1}} \bigg ) \bigg ) \end{aligned}$$

*for all *$$x \in V \backslash \{ 0 \}$$.

### *Proof*

First, we define $A := \{ f : V \rightarrow Y \,|\, f(0) = 0 \}$ and a mapping $J_m : A \rightarrow A$ by

$$\begin{aligned} J_m f(x) = a^{2m} f_e \bigg ( \frac{x}{a^m} \bigg ) + a^m f_o \bigg ( \frac{x}{a^m} \bigg ) \end{aligned}$$

for $x \in V$ and $m \in {\mathbb {N}}_0$. It follows from (9) that

18 $$\begin{aligned} \Vert J_m f(x) -&J_{m+l} f(x) \Vert \nonumber \\ \le \sum _{i=m}^{m+l-1}&\bigg \Vert a^{2i} f_e \bigg ( \frac{x}{a^i} \bigg ) + a^i f_o \bigg ( \frac{x}{a^i} \bigg ) - a^{2i+2} f_e \bigg ( \frac{x}{a^{i+1}} \bigg ) - a^{i+1} f_o \bigg ( \frac{x}{a^{i+1}} \bigg ) \bigg \Vert \nonumber \\ = \sum _{i=m}^{m+l-1}&\bigg \Vert a^{2i} \bigg ( f_e \bigg ( a \cdot \frac{x}{a^{i+1}} \bigg ) - a^2 f_e \bigg ( \frac{x}{a^{i+1}} \bigg ) \bigg ) \nonumber \\&\quad +a^i \bigg ( f_o \bigg ( a \cdot \frac{x}{a^{i+1}} \bigg ) - a f_o \bigg ( \frac{x}{a^{i+1}} \bigg ) \bigg ) \bigg \Vert \nonumber \\ \le \sum _{i=m}^{m+l-1}&\bigg ( a^{2i} \mu \bigg ( \frac{x}{a^{i+1}} \bigg ) + |a|^i \nu \bigg ( \frac{x}{a^{i+1}} \bigg ) \bigg ) \end{aligned}$$

for all $x \in V \backslash \{ 0 \}$. On account of (15) and (18), the sequence $\{ J_m f(x) \}$ is a Cauchy sequence for all $x \in V \backslash \{ 0 \}$. Since *Y* is complete and $f(0) = 0$, the sequence $\{ J_m f(x) \}$ converges for all $x \in V$. Hence, we can define a mapping $F : V \rightarrow Y$ by

$$\begin{aligned} F(x) := \lim _{m \rightarrow \infty } \bigg [ a^{2m} f_e \bigg ( \frac{x}{a^m} \bigg ) + a^m f_o \bigg ( \frac{x}{a^m} \bigg ) \bigg ] \end{aligned}$$

for all $x \in V$. Moreover, if we put $m = 0$ and let $l \rightarrow \infty$ in (18), we obtain the inequality (17).

In view of the definition of *F* and (10), we get the inequalities in (12) for all $x \in V$ and

$$\begin{aligned} \Vert DF&(x_1, x_2, \ldots , x_n) \Vert \\ =&\ \lim _{m \rightarrow \infty } \bigg \Vert a^{2m} Df_e \bigg ( \frac{x_1}{a^m}, \frac{x_2}{a^m}, \ldots , \frac{x_n}{a^m} \bigg ) + a^m Df_o \bigg ( \frac{x_1}{a^m}, \frac{x_2}{a^m}, \ldots , \frac{x_n}{a^m} \bigg ) \bigg \Vert \\ \le&\ \lim _{m \rightarrow \infty } \Bigg [ \frac{a^{2m}}{2} \bigg ( \varphi \bigg ( \frac{x_1}{a^m}, \frac{x_2}{a^m}, \ldots , \frac{x_n}{a^m} \bigg ) + \varphi \bigg ( \frac{-x_1}{a^m}, \frac{-x_2}{a^m}, \ldots , \frac{-x_n}{a^m} \bigg ) \bigg ) \\&\quad \qquad+\frac{|a|^{m}}{2} \bigg ( \varphi \bigg ( \frac{x_1}{a^m}, \frac{x_2}{a^m}, \ldots , \frac{x_n}{a^m} \bigg ) + \varphi \bigg ( \frac{-x_1}{a^m}, \frac{-x_2}{a^m}, \ldots , \frac{-x_n}{a^m} \bigg ) \bigg ) \Bigg ] \\ =&\ 0 \end{aligned}$$

for all $x_1, x_2, \ldots , x_n \in V \backslash \{ 0 \}$, *i.e.*, $DF(x_1, x_2, \ldots , x_n) = 0$ for all $x_1, x_2, \ldots , x_n \in V \backslash \{ 0 \}$. We notice that the equalities

$$\begin{aligned} F_e(|a|x) =&|a|^2 F_e(x),&F_e \bigg ( \frac{x}{|a|} \bigg ) = \frac{F_e(x)}{|a|^2}, \nonumber \\ F_o(|a|x) =&|a| F_o(x),&F_o \bigg ( \frac{x}{|a|} \bigg ) = \frac{F_o(x)}{|a|} \end{aligned}$$

hold in view of (12).

When $|a| > 1$, according to Lemma 1, there exists a unique mapping $F : V \rightarrow Y$ satisfying the equalities in (12) and the inequality (17), since the inequality

$$\begin{aligned} \Vert f&(x) - F(x) \Vert \\&\le \sum _{i=0}^{\infty } \bigg ( a^{2i} \mu \bigg ( \frac{x}{a^{i+1}} \bigg ) + |a|^i \nu \bigg ( \frac{x}{a^{i+1}} \bigg ) \bigg ) \\&= \sum _{i=0}^{\infty } \bigg ( a^{4i} \mu \bigg ( \frac{x}{a^{2i+1}} \bigg ) + a^{4i+2} \mu \bigg ( \frac{x}{a^{2i+2}} \bigg ) + a^{2i} \nu \bigg ( \frac{x}{a^{2i+1}} \bigg ) + |a|^{2i+1} \nu \bigg ( \frac{x}{a^{2i+2}} \bigg ) \bigg ) \\&\le \sum _{i=0}^{\infty } a^{4i} \phi \bigg ( \frac{x}{a^{2i}} \bigg ) \\&= \sum _{i=0}^{\infty } k^{2i} \phi \bigg ( \frac{x}{k^i} \bigg ) \end{aligned}$$

holds for all $x \in V$, where $k = a^2$ and $\phi (x) = \mu \big ( \frac{x}{a} \big ) + a^2 \mu \big ( \frac{x}{a^2} \big ) + \nu \big ( \frac{x}{a} \big ) + |a| \nu \big ( \frac{x}{a^2} \big )$.

When $|a| < 1$, according to Lemma 1, there exists a unique mapping $F : V \rightarrow Y$ satisfying the equalities in (12) and the inequality (17), since the inequality

$$\begin{aligned} \Vert f&(x) - F(x) \Vert \\&\le \sum _{i=0}^{\infty } \bigg ( a^{2i} \mu \bigg ( \frac{x}{a^{i+1}} \bigg ) + |a|^i \nu \bigg ( \frac{x}{a^{i+1}} \bigg ) \bigg ) \\&= \sum _{i=0}^{\infty } \bigg ( a^{4i} \mu \bigg ( \frac{x}{a^{2i+1}} \bigg ) + a^{4i+2} \mu \bigg ( \frac{x}{a^{2i+2}} \bigg ) + a^{2i} \nu \bigg ( \frac{x}{a^{2i+1}} \bigg ) + |a|^{2i+1} \nu \bigg ( \frac{x}{a^{2i+2}} \bigg ) \bigg ) \\&\le \sum _{i=0}^{\infty } a^{2i} \phi \bigg ( \frac{x}{a^{2i}} \bigg ) \\&= \sum _{i=0}^{\infty } \frac{\phi (k^i x)}{k^i} \end{aligned}$$

holds for all $x \in V$, where $k = \frac{1}{a^2}$ and $\phi (x) = \mu \big ( \frac{x}{a} \big ) + a^2 \mu \big ( \frac{x}{a^2} \big ) + \nu \big ( \frac{x}{a} \big ) + |a| \nu \big ( \frac{x}{a^2} \big )$. $\square$

### **Theorem 3**

*Let n be a fixed integer greater than 1, let *$\mu , \nu : V \backslash \{ 0 \} \rightarrow [0,\infty )$* be functions such that*

19 $$\begin{aligned} \begin{array}{ll} \left\{ \begin{array}{l} {\displaystyle \sum _{i=0}^\infty \frac{\mu (a^i x)}{a^{2i}} < \infty ,\qquad \sum _{i=0}^\infty \frac{\nu (a^i x)}{a^{2i}} < \infty ,} \\ {\displaystyle \sum _{i=0}^\infty |a|^i \mu \bigg ( \frac{x}{a^i} \bigg ) < \infty , \quad \sum _{i=0}^\infty |a|^i \nu \bigg ( \frac{x}{a^i} \bigg ) < \infty } \end{array} \right. &{} \quad { \text {when} \ }\quad |a| > 1,\\ \left\{ \begin{array}{l} {\displaystyle \sum _{i=0}^\infty a^{2i} \mu \bigg ( \frac{x}{a^i} \bigg ) < \infty , \quad \sum _{i=0}^\infty a^{2i} \nu \bigg ( \frac{x}{a^i} \bigg ) < \infty ,} \\ {\displaystyle \sum _{i=0}^\infty \frac{\mu (a^i x)}{|a|^i} < \infty , \qquad \sum _{i=0}^\infty \frac{\nu (a^i x)}{|a|^i} < \infty } \end{array} \right.&\quad { \text {when} \ }\quad |a| < 1 \end{array} \end{aligned}$$

*for all *$x \in V \backslash \{ 0 \}$*, and let *$\varphi : (V \backslash \{ 0 \})^n \rightarrow [0,\infty )$*be a function satisfying the conditions*

20 $$\begin{aligned} \begin{array}{ll} \left\{ \begin{array}{l} {\displaystyle \sum _{i=0}^\infty \frac{\varphi (a^i x_1, a^i x_2, \ldots , a^i x_n)}{a^{2i}} < \infty }, \\ {\displaystyle \sum _{i=0}^\infty |a|^i \varphi \bigg ( \frac{x_1}{a^i}, \frac{x_2}{a^i}, \ldots , \frac{x_n}{a^i} \bigg ) < \infty } \end{array} \right. &{} \quad { \text {when} \ }\quad |a| > 1, \\ \left\{ \begin{array}{l} {\displaystyle \sum _{i=0}^\infty \frac{\varphi (a^i x_1, a^i x_2, \ldots , a^i x_n)}{|a|^i} < \infty }, \\ {\displaystyle \sum _{i=0}^\infty a^{2i} \varphi \bigg ( \frac{x_1}{a^i}, \frac{x_2}{a^i}, \ldots , \frac{x_n}{a^i} \bigg ) < \infty } \end{array} \right.&\quad { \text {when} \ }\quad |a| < 1 \end{array} \end{aligned}$$

*for all *$x_1, x_2, \ldots , x_n \in V \backslash \{ 0 \}$*. If a mapping *$f : V \rightarrow Y$* satisfies *$f(0) = 0$* and the equality (*9*) for all *$x_1, x_2, \ldots , x_n \in V \backslash \{ 0 \}$*, then there exists a unique mapping *$F : V \rightarrow Y$* satisfying the equality (*11*) for all *$x_1, x_2, \ldots , x_n \in V \backslash \{ 0 \}$*, the equalities in (*12*) for all *$x \in V$*, and*

21 $$\begin{aligned} \Vert f(x) - F(x) \Vert \le \left\{ \begin{array}{ll} {\displaystyle \sum _{i=0}^{\infty } \bigg ( \frac{\mu (a^i x)}{a^{2i+2}} + |a|^i \nu \bigg ( \frac{x}{a^{i+1}} \bigg ) \bigg )} &{} \quad { \text {when} \ }\quad |a| > 1, \\ {\displaystyle \sum _{i=0}^{\infty } \bigg ( a^{2i} \mu \bigg ( \frac{x}{a^{i+1}} \bigg ) + \frac{\nu (a^i x)}{|a|^{i+1}} \bigg )} &{} \quad { \text {when} \ }\quad |a| < 1 \end{array} \right. \end{aligned}$$

*for all *$$x \in V \backslash \{ 0 \}$$.

### *Proof*

We will divide the proof of this theorem into two cases, one is for $|a| > 1$ and the other is for $|a| < 1$.

**Case 1** Assume that $|a| > 1$. We define a set $A := \{ f : V \rightarrow Y \,|\, f(0) = 0 \}$ and a mapping $J_m : A \rightarrow A$ by

$$\begin{aligned} J_m f(x) := \frac{f_e(a^m x)}{a^{2m}} + a^m f_o \bigg ( \frac{x}{a^m} \bigg ) \end{aligned}$$

for all $x \in V$ and $m \in {\mathbb {N}}_0$. It follows from (9) that

22 $$\begin{aligned} \Vert J_m f(x) -&J_{n+m} f(x) \Vert \nonumber \\ \le \sum _{i=m}^{m+l-1}&\bigg \Vert \frac{f_e (a^i x)}{a^{2i}} + a^i f_o \bigg ( \frac{x}{a^i} \bigg ) - \frac{f_e(a^{i+1}x)}{a^{2i+2}} - a^{i+1} f_o \bigg ( \frac{x}{a^{i+1}} \bigg ) \bigg \Vert \nonumber \\ = \sum _{i=m}^{m+l-1}&\bigg \Vert -\frac{f_e(a \cdot a^i x) - a^2 f_e(a^i x)}{a^{2i+2}} + a^i \bigg ( f_o \bigg ( a \cdot \frac{x}{a^{i+1}} \bigg ) - a f_o \bigg ( \frac{x}{a^{i+1}} \bigg ) \bigg ) \bigg \Vert \nonumber \\ \le \sum _{i=m}^{m+l-1}&\bigg ( \frac{\mu (a^i x)}{a^{2i+2}} + |a|^i \nu \bigg ( \frac{x}{a^{i+1}} \bigg ) \bigg ) \end{aligned}$$

for all $x \in V \backslash \{ 0 \}$. In view of (19) and (22), the sequence $\{ J_m f(x) \}$ is a Cauchy sequence for all $x \in V \backslash \{ 0 \}$. Since *Y* is complete and $f(0) = 0$, the sequence $\{ J_mf(x) \}$ converges for all $x \in V \backslash \{ 0 \}$. Hence, we can define a mapping $F: V \rightarrow Y$ by

$$\begin{aligned} F(x) := \lim _{m \rightarrow \infty } \bigg [\frac{f_e(a^m x)}{a^{2m}} + a^m f_o \bigg ( \frac{x}{a^m} \bigg )\bigg ] \end{aligned}$$

for all $x \in V$. Moreover, if we put $m = 0$ and let $l \rightarrow \infty$ in (22), we obtain the first inequality of (21). Using the definition of *F*, (10), and (20), we get the equalities in (12) for all $x \in V$ and

$$\begin{aligned} \Vert DF(x_1, x_2, \ldots , x_n) \Vert =&\ \lim _{m \rightarrow \infty } \bigg \Vert \frac{Df_e \big ( a^m x_1, \ldots , a^m x_n \big )}{a^{2m}} + a^m Df_o \bigg ( \frac{x_1}{a^m}, \ldots , \frac{x_n}{a^m} \bigg ) \bigg \Vert \\ \le&\ \lim _{m \rightarrow \infty } \Bigg [ \frac{\varphi \big ( a^m x_1, \ldots , a^m x_n \big ) + \varphi \big ( -a^m x_1, \ldots , -a^m x_n \big )}{2a^{2m}} \\&\quad \qquad +\frac{|a|^m}{2} \bigg ( \varphi \bigg ( \frac{x_1}{a^m}, \ldots , \frac{x_n}{a^m} \bigg ) + \varphi \bigg ( \frac{-x_1}{a^m}, \ldots , \frac{-x_n}{a^m} \bigg ) \bigg ) \Bigg ] \\ =&\ 0 \end{aligned}$$

for all $x_1, x_2, \ldots , x_n \in V \backslash \{ 0 \}$, *i.e.*, $DF(x_1, x_2, \ldots , x_n) = 0$ for all $x_1, x_2, \ldots , x_n \in V \backslash \{ 0 \}$. Notice that the equalities

$$\begin{aligned} F_e(|a|x) = |a|^2 F_e(x), \quad F_o(|a|x) = |a| F_o(x) \end{aligned}$$

are true in view of (12). Using Lemma 2, we conclude that there exists a unique mapping $F : V \rightarrow Y$ satisfying the equalities in (12) and the first inequality in (21), since the inequality

$$\begin{aligned} \Vert f(x) - F(x) \Vert \le&\ \sum _{i=0}^{\infty } \bigg ( \frac{\mu (a^i x)}{a^{2i+2}} + |a|^i \nu \bigg ( \frac{x}{a^{i+1}} \bigg ) \bigg ) \\ =&\ \sum _{i=0}^{\infty } \bigg ( \frac{a^2 \mu (a^{2i}x) + \mu (a^{2i}ax)}{a^{4i+4}} + a^{2i} \nu \bigg ( \frac{x}{a^{2i+1}} \bigg ) + |a|^{2i+1} \nu \bigg ( \frac{x}{a^{2i+2}} \bigg ) \bigg ) \\ \le&\ \sum _{i=0}^{\infty } \bigg ( \frac{\psi (k^i x)}{k^{2i}} + k^i \phi \bigg ( \frac{x}{k^i} \bigg ) \bigg ) \end{aligned}$$

holds for all $x \in V$, where $k = a^2$, $\psi (x) = \frac{a^2 \mu (x) + \mu (ax)}{a^4}$, and $\phi (x) = \nu \big ( \frac{x}{a} \big ) + |a| \nu \big ( \frac{x}{a^2} \big )$.

**Case 2** We now consider the case of $|a| < 1$ and define a mapping $J_m : A \rightarrow A$ by

$$\begin{aligned} J_m f(x) := a^{2m} f_e \bigg ( \frac{x}{a^m} \bigg )+ \frac{f_o(a^m x)}{a^m} \end{aligned}$$

for all $x \in V$ and $n \in {\mathbb {N}}_0$. It follows from (9) that

23 $$\begin{aligned} \Vert J_m f(x) -&J_{m+l} f(x) \Vert \nonumber \\ \le \sum _{i=m}^{m+l-1}&\bigg \Vert a^{2i} f_e \bigg ( \frac{x}{a^i} \bigg ) + \frac{f_o(a^i x)}{a^i} - a^{2i+2} f_e \bigg ( \frac{x}{a^{i+1}} \bigg ) - \frac{f_o(a^{i+1}x)}{a^{i+1}} \bigg \Vert \nonumber \\ = \sum _{i=m}^{m+l-1}&\bigg \Vert a^{2i} \bigg ( f_e \bigg ( a \cdot \frac{x}{a^{i+1}} \bigg ) - a^2 f_e \bigg ( \frac{x}{a^{i+1}} \bigg ) \bigg ) - \frac{f_o(a \cdot a^i x) - af_o(a^i x)}{a^{i+1}} \bigg \Vert \nonumber \\ \le \sum _{i=m}^{m+l-1}&\bigg ( a^{2i} \mu \bigg ( \frac{x}{a^{i+1}} \bigg ) + \frac{\nu (a^i x)}{|a|^{i+1}} \bigg ) \end{aligned}$$

for all $x \in V \backslash \{ 0 \}$. On account of (19) and (23), the sequence $\{ J_m f(x) \}$ is a Cauchy sequence for all $x \in V \backslash \{ 0 \}$. Since *Y* is complete and $f(0) = 0$, the sequence $\{J_mf(x) \}$ converges for all $x \in V$. Hence, we can define a mapping $F: V \rightarrow Y$ by

$$\begin{aligned} F(x) := \lim _{m \rightarrow \infty } \bigg [ a^{2m} f_e \bigg ( \frac{x}{a^m} \bigg ) + \frac{f_o(a^m x)}{a^m} \bigg ] \end{aligned}$$

for all $x \in V$. Moreover, if we put $m = 0$ and let $l \rightarrow \infty$ in (23), we obtain the second inequality in (21). From the definition of *F*, (10), and (20), we get the inequalities in (12) for all $x \in V$ and

$$\begin{aligned} \Vert DF&(x_1, x_2, \ldots , x_n) \Vert \\ =&\ \lim _{m \rightarrow \infty } \bigg \Vert a^{2m} Df_e \bigg ( \frac{x_1}{a^m}, \frac{x_2}{a^m}, \ldots , \frac{x_n}{a^m} \bigg ) + \frac{Df_o \big ( a^m x_1, a^m x_2, \ldots , a^m x_n \big )}{a^m} \bigg \Vert \\ \le&\ \lim _{m \rightarrow \infty } \Bigg ( \frac{a^{2m}}{2} \bigg ( \varphi \bigg ( \frac{x_1}{a^m}, \frac{x_2}{a^m}, \ldots , \frac{x_n}{a^m} \bigg ) + \varphi \bigg ( \frac{-x_1}{a^m}, \frac{-x_2}{a^m}, \ldots , \frac{-x_n}{a^m} \bigg ) \bigg ) \\&\quad \qquad +\frac{\varphi \big ( a^m x_1, a^m x_2, \ldots , a^m x_n \big ) + \varphi \big ( -a^m x_1, -a^m x_2, \ldots , -a^m x_n \big )}{2|a|^m} \Bigg ) \\ =&\ 0 \end{aligned}$$

for all $x_1, x_2, \ldots , x_n \in V \backslash \{ 0 \}$, *i.e.*, $DF(x_1, x_2, \ldots , x_n) = 0$ for all $x_1, x_2, \ldots , x_n \in V \backslash \{ 0 \}$. Notice that the equalities

$$\begin{aligned} F_e \bigg ( \frac{x}{|a|} \bigg ) = \frac{F_e(x)}{|a|^2} \quad { \text {and} \ }\quad F_o \bigg ( \frac{x}{|a|} \bigg ) = \frac{F_o(x)}{|a|} \end{aligned}$$

hold by considering (12).

Using Lemma 2, we conclude that there exists a unique mapping $F : V \rightarrow Y$ satisfying the equalities in (12) and the second inequality in (21), since the inequality

$$\begin{aligned} \Vert f(x) - F(x) \Vert \le&\ \sum _{i=0}^{\infty } \bigg ( a^{2i} \mu \bigg ( \frac{x}{a^{i+1}} \bigg ) + \frac{\nu (a^i x)}{|a|^{i+1}} \bigg ) \\ =&\ \sum _{i=0}^{\infty } \bigg ( a^{4i} \mu \bigg ( \frac{x}{a^{2i+1}} \bigg ) + a^{4i+2} \mu \bigg ( \frac{x}{a^{2i+2}} \bigg ) + \frac{\nu (a^{2i}ax)}{a^{2i+2}} + \frac{\nu (a^{2i}x)}{|a|^{2i+1}} \bigg ) \\ \le&\ \sum _{i=0}^{\infty } \bigg ( \frac{\psi (k^i x)}{k^{2i}} + k^i \phi \bigg ( \frac{x}{k^i} \bigg ) \bigg ) \end{aligned}$$

holds for $x \in V$, where $k = \frac{1}{a^2}$, $\psi (x) = \mu \big ( \frac{x}{a} \big ) + a^2 \mu \big ( \frac{x}{a^2} \big )$, and $\phi (x) = \frac{\nu (ax)}{a^2} + \frac{\nu (x)}{|a|}$. $\square$

We can replace $V \backslash \{ 0 \}$ with *V* in Theorems 1, 2, and 3.

### **Corollary 4**

*Let X be a normed space and let p,*$\theta$, $\delta$*, and *$\varepsilon$* be real constants such that *$p \not \in \{ 1, 2 \}$, $a \not \in \{ -1, 0, 1 \}$*, and *$\theta , \delta , \varepsilon > 0$*. If a mapping *$f : X \rightarrow Y$*satisfies *$f(0) = 0$,

24 $$\begin{aligned} \big \Vert f_e(ax) - a^2 f_e(x) \big \Vert \le \delta \Vert x \Vert ^p \quad { \ and \ }\quad \big \Vert f_o(ax) - a f_o(x) \big \Vert \le \varepsilon \Vert x \Vert ^p \end{aligned}$$

*for all *$x \in X \backslash \{ 0 \}$*, and the inequality*

25 $$\begin{aligned} \Vert D f(x_1, x_2, \ldots , x_n) \Vert \le \theta \big ( \Vert x_1 \Vert ^p + \Vert x_2 \Vert ^p + \cdots + \Vert x_n \Vert ^p \big ) \end{aligned}$$

*for all *$x_1, x_2, \ldots , x_n \in X \backslash \{ 0 \}$*, then there exists a unique mapping *$F : X \rightarrow Y$* such that (*11*) holds for all *$x_1, x_2, \ldots , x_n \in X \backslash \{ 0 \}$* and the equalities in (*12*) hold for all *$x \in X$*, as well as*

26 $$\begin{aligned} \Vert f(x) - F(x) \Vert \le \frac{\delta \Vert x \Vert ^p}{| a^2 - |a|^p |} + \frac{\varepsilon \Vert x \Vert ^p}{| |a| - |a|^p |} \end{aligned}$$

*holds for all *$x \in X \backslash \{ 0 \}$.

### *Proof*

If we put

$$\begin{aligned} \varphi (x_1, x_2, \ldots , x_n) := \theta \big ( \Vert x_1 \Vert ^p + \cdots + \Vert x_n \Vert ^p \big ) \end{aligned}$$

for all $x_1, x_2, \ldots , x_n \in X \backslash \{ 0 \}$, then $\varphi$ satisfies (8) when either $|a| > 1$ and $p < 1$ or $|a| < 1$ and $p > 2$, and $\varphi$ satisfies (12) when either $|a| > 1$ and $p > 2$ or $|a| < 1$ and $p < 1$. Moreover, $\varphi$ satisfies (15) when $1 < p < 2$. Therefore, by Theorems 1, 2, and 3, there exists a unique mapping $F : X \rightarrow Y$ such that (11) holds for all $x_1, x_2, \ldots , x_n \in X \backslash \{ 0 \}$, and (12) holds for all $x \in X$, and such that (26) holds for all $x \in X \backslash \{ 0 \}$. $\square$

## Applications

In this section, let $a \not \in \{ -1, 0, 1 \}$ be a rational constant, let $Df(x_1, x_2, \ldots , x_n) = 0$ be a quadratic-additive type functional equation, let $Af(x_1, x_2, \ldots , x_n) = 0$ be a Cauchy additive functional equation, and let $Qf(x_1, x_2, \ldots , x_n) = 0$ be a quadratic functional equation.

Assume that the functional equation $Df(x_1, x_2, \ldots , x_n) = 0$ is a quadratic-additive type functional equation. Then $F : V \rightarrow Y$ is a solution of the functional equation $Df(x_1, x_2, \ldots , x_n) = 0$ if and only if $F : V \rightarrow Y$ is a quadratic-additive mapping. If $F : V \rightarrow Y$ is a quadratic-additive mapping, then $F_e(x)$ and $F_o(x)$ are a quadratic mapping and an additive mapping, respectively. Hence $F_e(ax) = a^2 F_e(x)$ and $F_o(ax) = aF_o(x)$ for all $x \in V$, *i.e.*, *F* satisfies the equalities in (12).

Therefore, the following theorems follow from Theorems 1, 2, and 3.

### **Theorem 5**

*Let n be a fixed integer greater than 1, let *$\mu : V \rightarrow [0,\infty )$* be a function satisfying the condition (*7*) for all *$x \in V$*, and let *$\varphi : V^n \rightarrow [0,\infty )$* be a function satisfying the condition (*8*) for all *$x_1, x_2, \ldots , x_n \in V$*. If a mapping *$f : V \rightarrow Y$*satisfies *$f(0) = 0$*, (*9*) for all*$x \in V$*, and (*10*) for all *$x_1, x_2, \ldots , x_n \in V$*, then there exists a unique quadratic-additive mapping *$F : V \rightarrow Y$* such that the inequality (*13*) holds for all *$x \in V$.

### **Theorem 6**

*Let n be a fixed integer greater than 1, let *$\mu : V \rightarrow [0,\infty )$* be a function satisfying the condition (*15*) for all *$x \in V$*, and let *$\varphi : V^n \rightarrow [0,\infty )$* be a function satisfying the condition (*16*) for all *$x_1, x_2, \ldots , x_n \in V$*. If a mapping *$f : V \rightarrow Y$*satisfies *$f(0) = 0$*, (*9*) for all *$x \in V$*, and (*10*) for all *$x_1, x_2, \ldots , x_n \in V$*, then there exists a unique quadratic-additive mapping *$F : V \rightarrow Y$* such that the inequality (*17*) holds for all *$x \in V$.

### **Theorem 7**

*Let n be a fixed integer greater than 1, let *$\mu : V \rightarrow [0,\infty )$* be a function satisfying the condition (*19*) for all *$x \in V$*, and let *$\varphi : V^n \rightarrow [0,\infty )$* be a function satisfying the conditions (*20*) for all *$x_1, x_2, \ldots , x_n \in V$*. If a mapping *$f : V \rightarrow Y$*satisfies *$f(0) = 0$*, (*9*) for all *$x \in V$*, and (*10*) for all *$x_1, x_2, \ldots , x_n \in V$*, then there exists a unique quadratic-additive mapping *$F : V \rightarrow Y$*satisfying the inequality (*21*) for all *$x \in V$.

### **Corollary 8**

*Let X be a normed space and let *$p, \theta , \xi$* be real constants such that *$p \not \in \{ 1, 2 \}$, $a \not \in \{ -1, 0, 1 \}$*, and *$p, \xi , \theta > 0$*. If a mapping *$f : X \rightarrow Y$* satisfies (*24*) for all *$x \in X$* and the inequality (*25*) for all*$x_1, x_2, \ldots , x_n \in X$*, then there exists a unique quadratic-additive mapping *$F : X \rightarrow Y$* satisfying the inequality (*26*) for all *$x \in X$.

Assume that the functional equation $Qf(x_1, x_2, \ldots , x_n) = 0$ is a quadratic functional equation. Then $F : V \rightarrow Y$ is a solution of the functional equation $Qf(x_1, x_2, \ldots , x_n) = 0$ if and only if $F : V \rightarrow Y$ is a quadratic mapping. If $F : V \rightarrow Y$ is a quadratic mapping, then $F_e(x) = F(x)$ and $F_o(x) = 0$ for all $x \in V$. Hence, $F_e(ax) = F(ax) = a^2 F(x) = a^2 F_e(x)$ and $F_o(ax) = 0 = aF_o(x)$ for all $x \in V$, *i.e.*, *F* satisfies the equalities in (12). On the other hand, let the functional equation $Af(x_1, x_2, \ldots , x_n) = 0$ be a Cauchy additive functional equation. Then $F : V \rightarrow Y$ is a solution of the functional equation $Af(x_1, x_2, \ldots , x_n) = 0$ if and only if $F : V \rightarrow Y$ is an additive mapping. If $F : V \rightarrow Y$ is an additive mapping, then $F_e(x) = 0$ and $F_o(x) = F(x)$ for all $x \in V$. Hence, $F_e(ax) = 0 = a^2 F_e(x)$ and $F_o(ax) = F(ax) = aF(x) = aF_o(x)$ for all $x \in V$, *i.e.*, *F* satisfies the equalities in (12). Therefore, the following theorems are consequences of Theorems 5, 6, and 7.

### **Theorem 9**

*Let n be a fixed integer greater than 1, let *$\mu : V \rightarrow [0,\infty )$* be a function satisfying the condition (*7*) for all *$x \in V$*, and let *$\varphi : V^n \rightarrow [0,\infty )$* be a function satisfying the condition (*8*) for all *$x_1, x_2, \ldots , x_n \in V$*. If a mapping *$f : V \rightarrow Y$* satisfies *$f(0) = 0$*, (*9*) for all *$x \in V$*, and*

27 $$\begin{aligned} \Vert Qf(x_1, x_2, \ldots , x_n) \Vert \le \varphi (x_1, x_2, \ldots , x_n) \end{aligned}$$

*for all *$x_1, x_2, \ldots , x_n \in V$*, then there exists a unique quadratic mapping *$F : V \rightarrow Y$*such that the inequality (*13*) holds for all *$x \in V$.

### **Theorem 10**

*Let n be a fixed integer greater than 1, let *$\mu : V \rightarrow [0,\infty )$* be a function satisfying the condition (*15*) for all*$x \in V$*, and let *$\varphi : V^n \rightarrow [0,\infty )$*be a function satisfying the condition (*16*) for all *$x_1, x_2, \ldots , x_n \in V$*. If a mapping *$f : V \rightarrow Y$* satisfies *$f(0) = 0$*, (*9*) for all*$x \in V$*, and (*27*) for all *$x_1, x_2, \ldots , x_n \in V$*, then there exists a unique quadratic mapping *$F : V \rightarrow Y$*such that the inequality (*17*) holds for all *$x \in V$.

### **Theorem 11**

*Let n be a fixed integer greater than 1, let *$\mu : V \rightarrow [0,\infty )$* be a function satisfying the condition *(19) *for all *$x \in V$,* and let *$\varphi : V^n \rightarrow [0,\infty )$*be a function satisfying the conditions* (20) for all $x_1, x_2, \ldots , x_n \in V$.* If a mapping *$f : V \rightarrow Y$*satisfies *$f(0) = 0$, (9) *for all*$x \in V$, *and* (27) *for all*$x_1, x_2, \ldots , x_n \in V$, *then there exists a unique quadratic mapping *$F : V \rightarrow Y$*satisfying the inequality* (21) *for all*$x \in V$.

### **Corollary 12**

*Let X be a normed space and let *$p, \theta , \xi$*be real constants such that*$p \not \in \{ 1, 2 \}$, $a \not \in \{ -1, 0, 1 \}$, *and*$p, \xi , \theta > 0$. *If a mapping*$f : X \rightarrow Y$*satisfies* (24) *for all*$x \in X$*and the inequality*

$$\begin{aligned} \Vert Qf(x_1, x_2, \ldots , x_n) \Vert \le \theta \big ( \Vert x_1 \Vert ^p + \Vert x_2 \Vert ^p + \cdots + \Vert x_n \Vert ^p \big ) \end{aligned}$$

*for all*$x_1, x_2, \ldots , x_n \in X$, *then there exists a unique quadratic mapping*$F : X \rightarrow Y$*satisfying the inequality* (26) for all $x \in X$.

### **Theorem 13**

*Let n be a fixed integer greater than 1, let *$\mu : V \rightarrow [0,\infty )$*be a function satisfying the condition *(7) *for all*$x \in V$, *and let*$\varphi : V^n \rightarrow [0,\infty )$*be a function satisfying the condition* (8) *for all*$x_1, x_2, \ldots , x_n \in V$.* If a mapping*$f : V \rightarrow Y$*satisfies*$f(0) = 0$, (9) *for all*$x \in V$, *and*

28 $$\begin{aligned} \Vert Af(x_1, x_2, \ldots , x_n) \Vert \le \varphi (x_1, x_2, \ldots , x_n) \end{aligned}$$

*for all*$x_1, x_2, \ldots , x_n \in V$, *then there exists a unique additive mapping*$F : V \rightarrow Y$*such that the inequality* (13) *holds for all*$x \in V$.

### **Theorem 14**

*Let n be a fixed integer greater than 1, let*$\mu : V \rightarrow [0,\infty )$*be a function satisfying the condition* (15) *for all*$x \in V$, *and let*$\varphi : V^n \rightarrow [0,\infty )$*be a function satisfying the condition* (16) *for all*$x_1, x_2, \ldots , x_n \in V$. *If a mapping*$f : V \rightarrow Y$*satisfies*$f(0) = 0$, (9) *for all*$x \in V$, *and* (28) *for all*$x_1, x_2, \ldots , x_n \in V$, *then there exists a unique additive mapping*$F : V \rightarrow Y$*such that the inequality* (17) *holds for all*$x \in V$.

### **Theorem 15**

*Let n be a fixed integer greater than 1, let *$\mu : V\rightarrow [0,\infty )$*be a function satisfying the condition* (19) *for all*$x \in V$, *and let*$\varphi : V^n \rightarrow [0,\infty )$*be a function satisfying the conditions* (20)* for all*$x_1, x_2, \ldots , x_n \in V$.* If a mapping*$f : V \rightarrow Y$*satisfies*$f(0) = 0$, (9) *for all*$x \in V$, *and* (28*) for all *$x_1, x_2, \ldots , x_n \in V$*, then there exists a unique additive mapping *$F : V \rightarrow Y$* satisfying the inequality (*21*) for all *$x \in V$.

### **Corollary 16**

*Let X be a normed space and let *$p, \theta , \xi$* be real constants such that *$p \not \in \{ 1, 2 \}$, $a \not \in \{ -1, 0, 1 \}$*, and *$p, \xi , \theta > 0$*. If a mapping *$f : X \rightarrow Y$* satisfies (*24*) for all *$x \in X$* and the inequality*

$$\begin{aligned} \Vert Af(x_1, x_2, \ldots , x_n) \Vert \le \theta \big ( \Vert x_1 \Vert ^p + \Vert x_2 \Vert ^p + \cdots + \Vert x_n \Vert ^p \big ) \end{aligned}$$

*for all *$x_1, x_2, \ldots , x_n \in X$*, then there exists a unique additive mapping *$F : X \rightarrow Y$*satisfying the inequality (*26*) for all *$x \in X$.

## Conclusions

The conditions (8) and (10) are given in the most stability theorems, and we try to prove (11) and (13) for the generalized Hyers-Ulam stability. Unfortunately, their proofs are usually long and tedious.

However, if we confine ourselves to the stability problems of the quadratic-additive type functional equations, then the condition (12) is a direct consequence of (11). Therefore, according to Theorem 1, it only needs to prove the conditions (7) and (9) by using (8) and (10) for the generalized Hyers-Ulam stability of these equations. In many practical applications, it is an easy thing to show that (7) and (9) are true provided the assumptions (8) and (10) are given.

In this way, we significantly simplify the proof for the stability of quadratic-additive type functional equations. Hence, Theorem 1 has the strong advantage of other stability theorems. The same things are valid for the other main theorems of this paper, Theorems 2 and 3.
